# Dynamical control on helicity of electromagnetic waves by tunable metasurfaces

**DOI:** 10.1038/srep27503

**Published:** 2016-06-08

**Authors:** He-Xiu Xu, Shulin Sun, Shiwei Tang, Shaojie Ma, Qiong He, Guang-Ming Wang, Tong Cai, Hai-Peng Li, Lei Zhou

**Affiliations:** 1 State Key Laboratory of Surface Physics, Key Laboratory of Micro and Nano Photonic Structures (Ministry of Education) and Physics Department, Fudan University, Shanghai 200433, China; 2 Air and Missile Defense College, Air force Engineering University, Xi’an, 710051, China; 3 Shanghai Engineering Research Center of Ultra-Precision Optical Manufacturing, Green Photonics and Department of Optical Science and Engineering, Fudan University, Shanghai 200433, China; 4 Department of Physics, Faculty of Science, Ningbo University, Ningbo 315211, China; 5 Collaborative Innovation Center of Advanced Microstructures, Fudan University, Shanghai 200433, China

## Abstract

Manipulating the polarization states of electromagnetic (EM) waves, a fundamental issue in optics, attracted intensive attention recently. However, most of the devices realized so far are either too bulky in size, and/or are passive with only specific functionalities. Here we combine theory and experiment to demonstrate that, a tunable metasurface incorporating diodes as active elements can dynamically control the reflection phase of EM waves, and thus exhibits unprecedented capabilities to manipulate the helicity of incident circular-polarized (CP) EM wave. By controlling the bias voltages imparted on the embedded diodes, we demonstrate that the device can work in two distinct states. Whereas in the “On” state, the metasurface functions as a helicity convertor and a helicity hybridizer within two separate frequency bands, it behaves as a helicity keeper within an ultra-wide frequency band in the “Off” state. Our findings pave the way to realize functionality-switchable devices related to phase control, such as frequency-tunable subwavelength cavities, anomalous reflectors and even holograms.

Polarization is an important characteristic of electromagnetic (EM) wave^1^, and manipulating the polarization state of EM wave is crucial in photonic research. Conventional devices, such as optical gratings and dichroic crystals, suffer the issue of low working efficiency and/or are too bulky in size^2^, especially for long-wavelength applications. Metamaterials, artificial materials constructed by subwavelength microstructures with tailored EM properties, attracted lots of attention recently since they exhibit unprecedented capabilities to manipulate EM waves^3^. In particular, many fascinating polarization-manipulation effects, such as polarization conversion and rotation, have been realized by carefully designed metamaterials^4^,^5^,^6^,^7^,^8^,^9^,^10^,^11^,^12^,^13^,^14^,^15^,^16^ or metasurfaces^1^,^17^,^18^,^19^,^20^,^21^,^22^,^23^,^24^,^25^,^26^,^27^,^28^,^29^ (ultrathin metamaterials constructed by planar EM resonators) with anisotropic or chiral EM responses, at frequencies ranging from microwave to optics. These meta-devices are typically much thinner than wavelength and can exhibit very high polarization-manipulation efficiencies. However, most of these polarization-manipulation meta-devices^4^,^5^,^6^,^7^,^8^,^9^,^10^,^11^,^12^,^13^,^14^,^15^,^16^,^17^,^18^,^19^,^20^,^21^,^22^,^23^,^24^,^25^,^26^,^27^,^28^,^29^ are passive, which means that once they are fabricated their functionalities cannot be changed. In practical applications, it is highly desirable to have polarization manipulators whose functionalities can be dynamically *tuned* by an external “knob”. Although several approaches have been proposed to actively tune the EM responses of metamaterials in different frequency domain (such as tuning the resonance frequency^30^,^31^,^32^,^33^, EM wave-front^34^,^35^,^36^,^37^,^38^,^39^, and transmission or reflection properties^40^,^41^), applying these approaches to realize dynamic polarization-related devices with multifunctionalities are rarely seen.

In this paper, we combine theory and experiment to demonstrate an active polarization-manipulation meta-device, in which different functionalities are integrated into one single tunable metasurface (TMS), including circularly-polarized (CP) helicity convertor, helicity hybridizer and helicity keeper. Our tunable device is realized by combining a passive metasurface with active positive intrinsic-negative (PIN) diodes. The underlying physics is that connecting/disconnecting the diode can significantly modulate the EM response (in particular, the reflection phases) of the metasurface via changing its resonance frequency^40^, thereby switch the functionality of the meta-device dramatically. Such a mechanism is fundamentally different from the loss-driven underdamped to overdamped resonator transition proposed recently^41^,^42^, since here the reflection amplitude remains nearly unchanged. As a particular demonstration, we design and fabricate a TMS in which a PIN dipole is incorporated into the unit cell, and experimentally demonstrate that the polarization-manipulation functionality of the device can be dynamically modulated when the diodes are biased at two appropriate voltages. Our results pave the road to make other tunable and functional devices related to dynamical phase control, such as tunable subwavelength cavities, anomalous reflectors and even animated holograms. As an example, we employ full-wave simulations to design a frequency-tunable resonant cavity based on our TMS in the end of this paper.

## Results

### Criterions for dynamical control on helicity

It is well known that a CP wave will change its helicity after it is reflected back by a conventional mirror. Although in many applications such helicity reversal does not cause substantial problems, in some other cases such as satellite communications, radar detections, etc., it does represent an issue which should be properly solved. Therefore, it is highly desirable to have a tunable device that can dynamically switch its functionality on helicity control of CP waves.

We first consider the criterions to realize certain helicity manipulations by a reflective metasurface. Consider a CP wave with E-field component 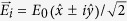 normally incident on a metasurface placed on the *xy* plane. Suppose the incident wave is propagating along the *z* direction, then its polarization is left/right circular polarization (L/RCP) if the sign in front of 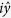 is 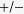
+/−, under the time-harmonic convention 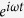. Assuming that the metasurface exhibits mirror symmetries with respect to the *yz* and *xz* planes, the reflected EM wave can be generally expressed as


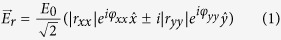


where 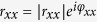 and 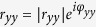 are the reflection coefficients for EM waves polarized along two symmetry axes. To ensure that the reflected wave is still circularly polarized, we require that


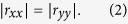


**Figure 1:**
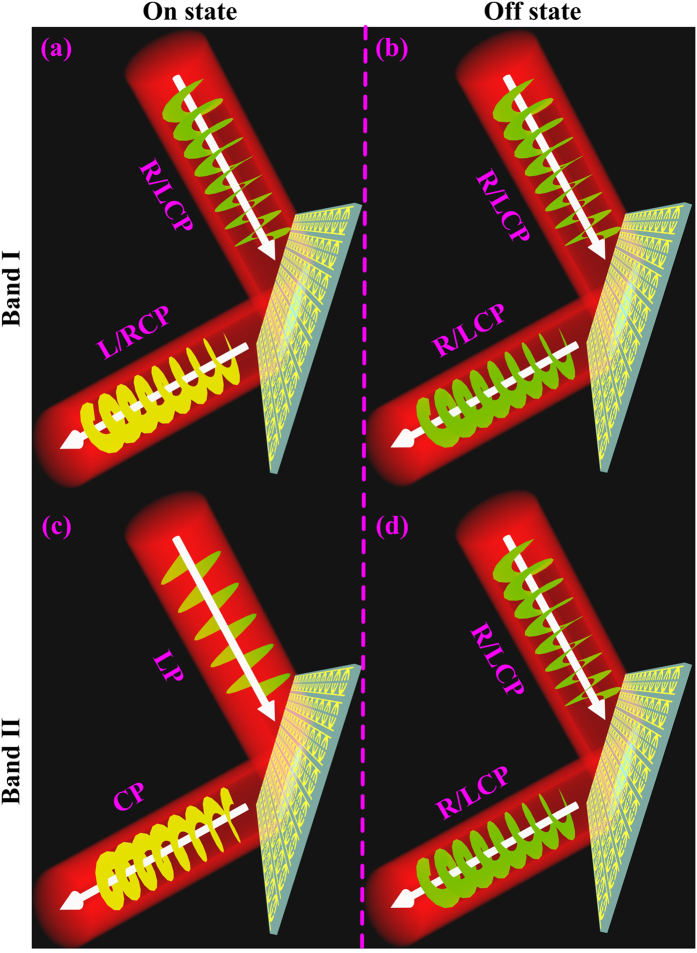
Schematics of the proposed multifunctional TMS. The functionalities of the TMS switch between helicity convertor and helicity keeper in band I (**a,b**) and between LP polarizer and helicity keeper in band II **(c,d**). Here, the TMS works in either the “On” state (**a,c**) or the “Off” state (**b,d**).

In addition, since the wave-vector of the EM wave is reversed after the reflection, we immediately understand that the handness of the reflected CP wave remains identical to that of the incident one (see Fig. 1(b)) only if the condition is satisfied.


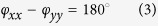


On the other hand, the helicity of reflected CP wave will be reversed (see Fig. 1(a)) if the phases satisfy


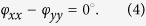


Note that here the phases are all limited to the branch of [−180°, 180°] without causing any confusions. Finally, when the condition


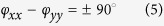


is fulfilled, the reflected wave will become a linearly-polarized (LP) beam containing equal amounts of RCP and LCP components, see Fig. 1(c).

Eqs (2)–(5) show that a metasurface can have diversified helicity-manipulation functionalities if its reflection phases 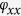 and 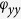 satisfy certain relations. Therefore, we can design a TMS to dynamically control the helicity of EM waves simply by making its 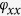 and 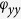 satisfy certain desired relations (e.g., Eqs (2)–(5), 24, 10, 11). As an illustration of this general idea, here we present one particular tunable device among many other possibilities. Figure 1 schematically depicts what we want to achieve with our device: the meta-device behaves as a helicity convertor and a helicity hybridizer for input CP wave within two separate frequency bands in the “On” state, but becomes a helicity keeper (i.e., helicity-conserved reflector) within an ultra-wide band in the “Off” state. In what follows, we describe how to realize such a device.

### Topology, equivalent circuit model and design

**Figure 2:**
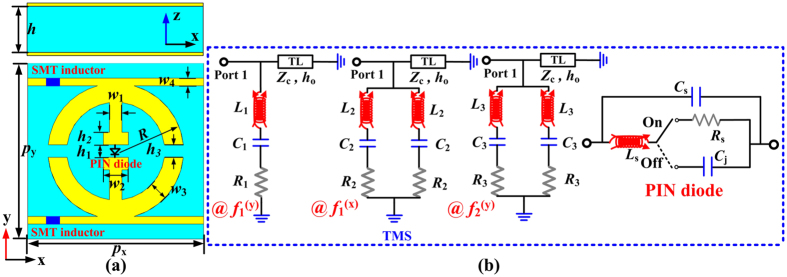
Topology and equivalent circuit models of the building block of the designed TMS. (**a**) Side and top views of the building block. (**b**) Equivalent circuit models of the TMS at resonance frequencies *f*_1_^(y)^, *f*_2_^(y)^ for y-polarization excitation and *f*_1_^(x)^ for x-polarization excitation. Here, *p*_x_ = *p*_y_ = 14 mm, *w*_1_ = 1 mm, *w*_2_ = 2 mm, *w*_3_ = 1.5 mm, *w*_4_ = 0.6 mm, *R* = 5.4 mm, *h* = 6 mm, *h*_1_ = 1 mm, *h*_2_ = 1 mm, *h*_3_ = 1 mm, and *L*_j_ = 10 nH.

To realize the functionality-switching effects depicted in Fig. 1, we design a TMS whose 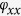 and 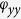 can be controlled by active elements incorporated in its unit cell. As shown in Fig. 2(a), our unit cell consists of a metallic electric inductive-capacitive resonator (ELC) coupled with a perfect-electric-conductor (PEC) ground plane through a dielectric spacer. The ground plane ensures that the device is totally reflective without any transmission. The ELC is basically a topological variant of split-ring resonator with geometrical parameters shown in Fig. 2(b). To electrically tune the EM response of the ELC, we connect the two central metallic wires in the ELC resonator by a PIN diode (SMP1345-079LF, Skyworks Solutions Inc^43^), which can be biased at a given voltage using two high-impedance lines that have perfect electrical connections with the ELC resonator. To achieve the desired reflection phase and to prevent the microwave signal from entering the bias line, we adopted a Murata LQW04AN10NH00 chip inductor with inductance *L*_j_ = 10 nH and self-resonance frequency higher than 7 GHz. Applying two appropriate DC voltages on the PIN diode, we can set it working in “On” or “Off” states and thus change the EM responses of the TMS dramatically (see Section 1 in the Supplementary Information for more discussions on the design strategy). Indeed, the TMS shown in Fig. 2(a) can be modeled by appropriate equivalent circuit models (CM) depicted in Fig. 2(b), at three resonance frequencies. More detailed discussions can be found in Section 2 of the Supplementary Information.

Now let us utilize the criterion presented in last section to design the dynamic helicity modulator with an optimum bandwidth based on the transmission line (TL) theory. As a starting point, the *ABCD* matrix of the TMS structure as well as the equivalent TL can be expressed as


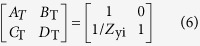



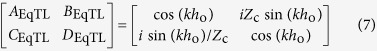


where *k* is the equivalent wave vector of the TEM wave, and *Z*_*y*i_ is the impedance of the shunt branch at three resonances and can be calculated as 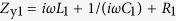, 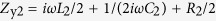 and 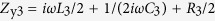, respectively. The total matrix can be readily calculated by cascading the above two *ABCD* matrices, yielding


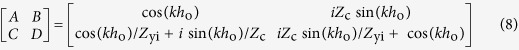


then the S parameters with phase information can be immediately achieved from the *ABCD* matrix by a simple transformation. These analytic expressions are tedious and are not presented here. To obtain a wide operation bandwidth, we require that the reflection phases for two orthogonal polarizations exhibit similar slopes as frequency varies, so that their difference can remain nearly a constant within a wide frequency band. Therefore, we enforce the following condition


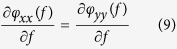


at two specific frequencies 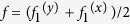 and 
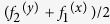
 in designing our structure. Here, 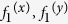 and 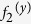 are three resonance frequencies which will be explained below. In our CM simulations, the lumped parameters are retrieved in Agilent’s Advanced Design System (ADS) from EM simulated reflection magnitude and phase. The CM simulated LP reflection coefficients are then utilized to calculate the CP reflection coefficients using 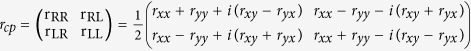. The lumped parameters are retrieved as *L*_1_ = 30 nH, *C*_1_ = 0.107 pF, *R*_1_ = 10.9 Ω, *Z*_c_ = 368.3 Ω and *k* * *h*_o_ = 44.8^o^ ranging from 2 to 5 GHz in the “On” state, and as *L*_1_ = 12.7 nH, *C*_1_ = 0.064 pF, *R*_1_ = 3.87 Ω, *Z*_c_ = 371 Ω and *k* * *h*_o_ = 38.4^o^ ranging from 2 to 5.4 GHz in the “Off” state. As expected, one can see that *C*_1_ and *L*_1_ are significantly altered by the diode while all the others parameters are nearly unaffected between the “On” and “Off” state. These retrieved circuit parameters will be substituted into Eq. (9) to check the effectiveness of the design.

### Simulation and measurement results

**Figure 3:**
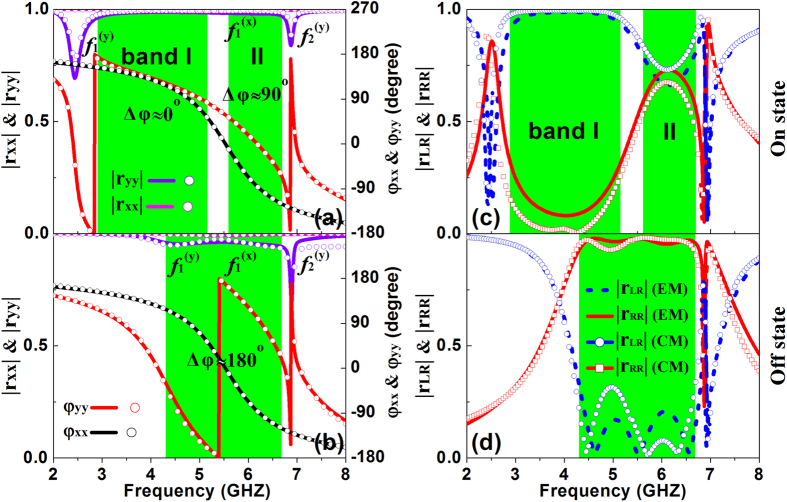
Electromagnetic responses of the designed TMS. Calculated LP reflection coefficients (**a,b**) and CP reflection coefficients (**c,d**) of the designed TMS in the “On” (**a,c**) and ”Off” (**b,d**) states based on FDTD (lines) and CM simulation (symbols).

Lines in Fig. 3(a,b) are the reflection coefficients of the TMS in the “On” and “Off” states, respectively, obtained by finite-difference-time-domain (FDTD) simulations. When the device is in the “On” state (i.e., the PIN diode is forward biased), two resonant modes (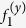
≈2.43 GHz and 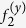 = 6.88 GHz) can be identified from the calculated |*r*_yy_| spectrum, while only a weak resonant dip (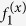
≈5.67 GHz) is found from the |*r*_xx_| spectrum (Fig. 3a). Meanwhile, *φ*_yy − _*φ*_xx_ remains nearly 0° within 2.9~5.13 GHz (Band I) and nearly 90° within 5.58~6.69 GHz (Band II), implying that the device functions as a helicity converter in Band I and as a helicity hybridizer in Band II, respectively. Note that the reflection amplitudes |*r*_yy_| and |*r*_xx_| are both nearly 1 within these two frequency bands. When the device is in the “Off” state, however, its EM responses change drastically (see Fig. 3b). Now the low-frequency resonant mode for y-polarization (i.e., 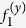) is shifted to a new position 4.6 GHz, while the other two modes are nearly unaffected. As a result, the *φ*_yy_ spectrum is modified significantly while the *φ*_xx_ spectrum remains nearly unchanged after the “On-Off” switching. In such a case, *φ*_yy_ − *φ*_xx_ now stays ~180^o^ within a broad frequency range (4.33–6.63 GHz, Fig. 3b), implying that the device keeps the helicity of incident CP wave after reflection. We note that |*r*_yy_| and |*r*_xx_| are again nearly 1 within such a band. Such functionality-switching can be more clearly seen from Fig. 3(c,d) where the FDTD simulated helicity-conserved and helicity-reversed reflectance spectra are shown for the device working in the “On” and “Off” states, respectively. Clearly, the device functions as a helicity convertor in Band I and a helicity hybridizer in Band II when it is in the “On”-state (Fig. 3(c)), but becomes a CP helicity keeper within a new band (4.33~6.63 GHz) when it is in “Off” state. In both states, the polarization extinction ratio for the helicity conversion and keeping is more than 10 dB, see Fig. S7 in Supplementary Information.

**Figure 4:**
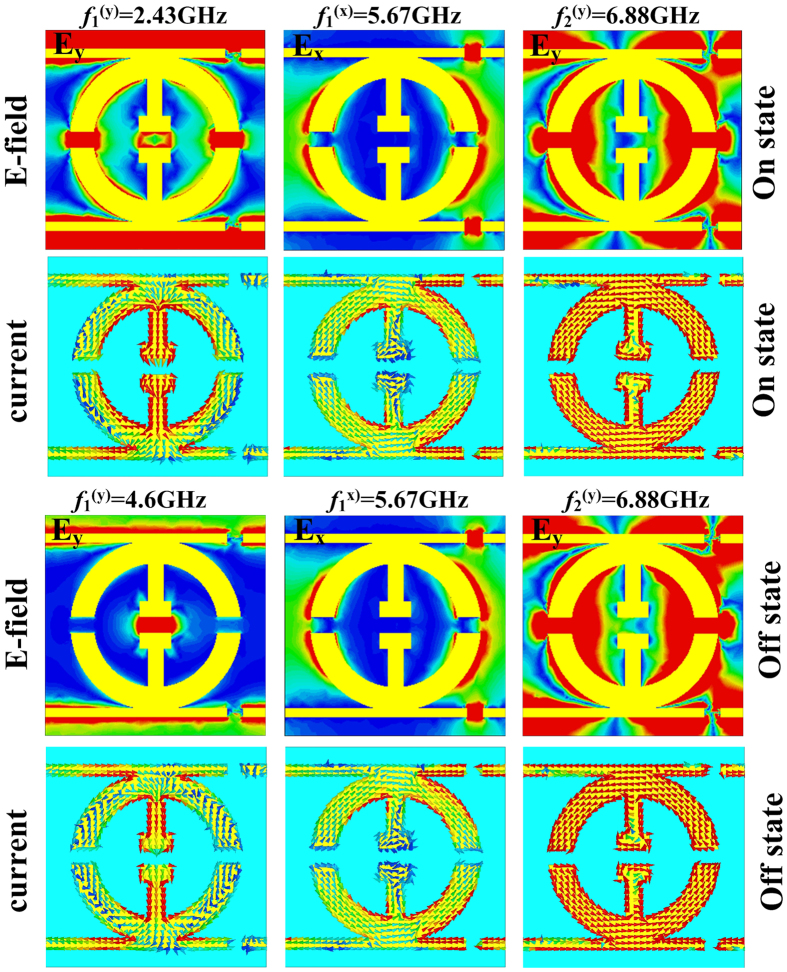
The calculated electric field and surface current distributions at three resonance frequencies of the designed TMS.

To understand the physics underlying such functionality-switching behavior, we studied the current/field distributions of three resonant modes in our structures. Consider the two *y*-polarization modes first. While in the 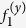 mode the electric currents are excited to flow in two central vertical lines and two arcs, they are mainly localized along two arcs in the 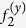 mode (Fig. 4). Meanwhile, the *x*-polarization mode 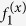 is also predominately associated with the two arcs of the resonant structure, but has nothing to do with the central vertical lines. The nature of these resonant modes helps us understand the principle of the dynamical switching. Since only the 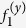 mode is connected with the central vertical lines, using a PIN diode to short-circuit the central gap can thus significantly modify such a resonant mode, but has nearly no effects on the other two modes (see Fig. 3(a,b)). In particular, when the PIN diode is working to connect two central lines (i.e., in the “On” state), the associated resonant structure exhibits a longer metallic length and the induced currents flow in both the central vertical lines and the two arcs. Conversely, while the PIN diode is working to disconnect two central lines (i.e., in the “Off” state), the associated resonant structure exhibits a shorter metallic length, which explains why the resonance frequency is significantly blue-shifted. Therefore, the working principle of our device is clear: Using a PIN diode to control the resonant frequency of one of the resonant modes, we can dynamically manipulate the phase difference 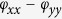, and in turn, the capability to modulate the helicity of incident CP wave. We emphasize that such a mechanism is fundamentally different from that proposed in refs [37 and 38] where loss plays very important roles.

More insights can be obtained by checking the equivalent CM of the proposed TMS. As already discussed, the ELC structure possesses two *y*-polarized resonant modes and one *x*-polarized mode. Knowledges on these resonant modes help us establish their corresponding equivalent CMs (Fig. 2(b)). The role of the PIN diode is thus clear. The central gap will be connected or disconnected when the PIN diode works in the “On” and “Off” state, respectively, which dynamically controls the resonant frequency 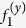 via changing the capacitance C and inductance L of the related circuit. In contrast, the other two resonances are nearly unaffected by the PIN diode. To achieve an optimum bandwidth for the proposed device, in real design we fine-tuned our structure to make 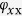 and 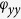 exhibit similar slopes around the central working frequency in the “Off” state, see established criterion in previous section. Results calculated with the equivalent CMs are shown as symbols in Fig. 3, which are in good agreement with full-wave simulation results.

**Figure 5:**
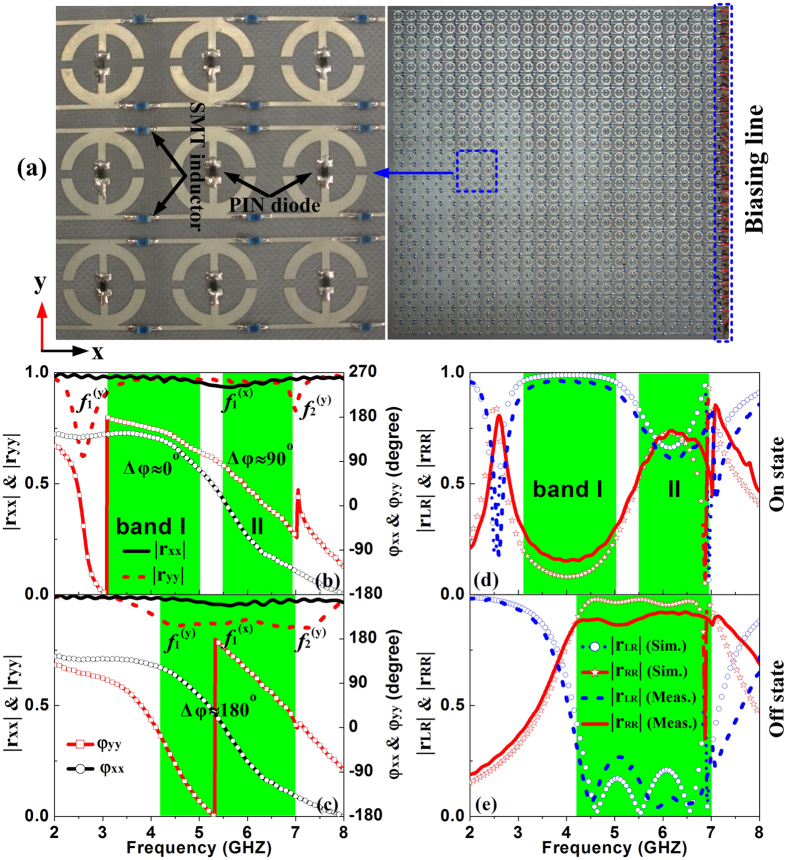
Picture and the measured LP and CP reflection coefficients of the fabricated TMS. (**a**) The building block of the TMS are connected by the SMT inductors along x –direction with the biasing line to control the voltage of PIN diode. (**b,c**) In LP basis, the reflection phase difference between *φ*_yy_ and *φ*_xx_ is kept at about 0^o^ in Band I (3.11~5.01GHz) and about 90° in Band II (5.51~6.94 GHz) in the “On” state and is kept at about 180° in an ultra-wide band (4.21~7.01 GHz) in “Off” state. (**d,e**) In CP basis, the TMS functions as a CP helicity convertor in Band I and CP helicity hybridizer (i.e., CP polarizer) in Band II in the “On” state, and the CP helicity keeper in the ultra-wide band (4.21~7.01 GHz).

We fabricated a TMS according to our design and experimentally characterized its EM responses (see Methods). The sample consists of 25 × 25 elements embedded with 625 diodes and 1350 Murata inductors and its total size is 360 × 360 mm^2^ (see Fig. 5(a)). Elements in the same line along *x* direction are connected to each other, so that they automatically have the same biasing voltage. On the other hand, elements in different horizontal lines are independent and thus their biasing voltages should be carefully adjusted. We note that such a configuration offers us the exciting possibility to independently engineer the phases in different horizontal lines so that the final phase profile can exhibit a linear or parabolic distribution, which can be very useful in other beam-manipulation applications. In this paper, however, we focus on the situation that all elements are tuned to exhibit the same phase response. Fig. 5(b,c) depict the experimentally measured reflection coefficients of the fabricated sample, which are in reasonable agreement with FDTD simulation results in Fig. 3. The slight differences between simulated and measured spectra are primarily due to the additional resistances from the soldering pads, and the misalignment of the source/receiver antennas. As expected, three resonant dips appear at 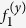 = 2.6 GHz, 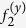 = 6.99 GHz and 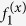 = 5.58 GHz when the device is in the “On” state, and their positions are shifted to 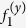 = 4.65 GHz, 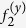 = 7.03 GHz and 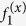 = 5.62 GHz when the device is in the “Off” state. In addition, the shallow dip and slowly dispersed phase response at 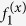 indicate that such a mode exhibits a low quality factor, as expected. However, compared with the FDTD spectra (Fig. 3), now the measured reflection phase only covers a small region for the mode 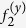. This can be attributed to the enhanced absorption in real sample which destroys the high-quality-factor resonance, see Section 4 in the Supplementary Information for more discussions. Nevertheless, such deviations do not significantly affect the functionality of our device. Indeed, as shown in Fig. 5(d,e) where the reflection spectra in terms of CP bases are re-plotted, the functionality of our device changes significantly as we turn “On/Off” the diode, as expected.

### Other potential applications

**Figure 6:**
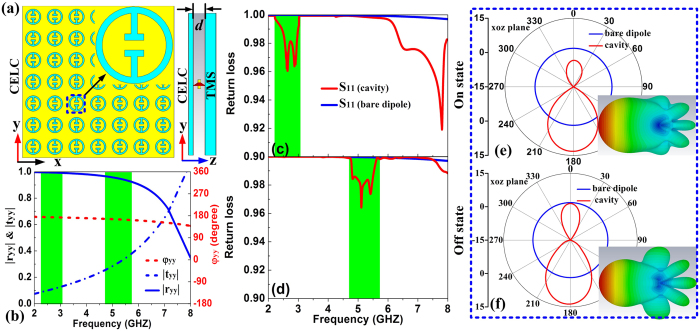
The schematics, reflection coefficients and resonance modes of the frequency-tunable subwavelength cavity. (**a**) The schematics of the cavity consisting of CELC and TMS. (**b**) Scattering coefficients of the CELC, (**c,d**) return loss spectrum and (**e,f**) radiation pattern of the subwavelength cavity in the “On” and “Off” state. The lateral size of both CELC and TMS plate is 182 mm × 182 mm in the “On” state while 98 mm × 98 mm in the “Off” state. The geometrical parameters of the CELC structure are *p*_x_ = *p*_y_ = 14 mm, *w*_1_ = *w*_3_ = *h*_2_ = 1 mm, *w*_2_ = 3 mm, *R* = 5 mm and *h* = *h*_1_ = 1.5 mm.

Our TMS can find many other interesting applications, which will be briefly introduced in this section. For example, in previous discussions we only used the diode to control one of the resonant modes. Obviously, more fascinating functionalities can be realized if we choose to control two or three resonant modes simultaneously. In addition, here we only utilized the Δ*φ* degree of freedom to achieve the polarization modulation, but in fact individually tuning *φ*_yy_ or *φ*_xx_ can also find many interesting applications. As schematically shown in Fig. 6(a), we can combine a TMS with a passive reflector to form a double-plate resonant cavity. According to ref. 44, now the resonance frequencies of such a metamaterial-based cavity are determined by


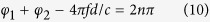


where 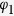 and 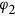 are respectively the reflection phases of EM waves at the TMS and the passive reflector, 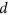 is the distance between two plates, and *n* is an arbitrary integer. Different from a conventional cavity, now 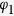 of our TMS can be dynamically controlled by switching on/off the diode (see Fig. 5(c)), and thus the resonance frequencies of our cavities can also be dynamically controlled. In addition, the fact that 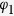 of our TMS covers the whole 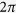 phase range implies that the size of our cavity can break the half-wavelength restriction imposed on conventional cavities^44^.

We used FDTD simulations to demonstrate the feasibility of the proposed idea. In our design, the passive reflector consists of complementary ELCs (CELCs) printed on a F4B dielectric substrate (thickness *h* = 1.5 mm, *ε*_*r*_ = 2.65 with loss tangent of 0.001), and the cavity thickness is set as d = 4 mm. We first studied the reflection/transmission spectra of the passive reflector. Figure 6(b) shows that the passive reflector is highly reflective for *y*-polarized EM waves with reflection phase staying around 180^o^ in the frequency range of interest (2–7 GHz), indicating that it is just a conventional electric reflector. To probe the resonant modes of our cavity, we put a *y*-oriented 6mm-long dipole antenna into the cavity and use FDTD simulations to study its radiation properties. Fig. 6(c,d) show that the S11 spectra of the antenna are dramatically changed after it is placed into the cavity working in “On” and “Off” states, respectively. In particular, the new pronounced dips (around 2.62 GHz in “On” state, and around 4.86 GHz in “Off” state) in S11 spectra are the signature of the resonance modes, since such a short antenna does not radiate efficiently at these frequencies (see blue curve for S11 spectra of the bare dipole antenna). We note that the thickness of our cavity is only about λ_0_/30 and λ_0_/15 at these two resonance frequencies, indicating that our cavity is deeply subwavelength. As a comparison, the lowest resonance mode is at 37.5 GHz for a conventional double-plate cavity with the same thickness. Most importantly, the working frequency band of our subwavelength cavity is dynamically switched by controlling the working state of the PIN diodes incorporated in the TMS. The underlying physics is that, turning on/off the PIN diode can dynamically control 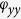 of the TMS, and in turn, switch the working frequencies of the resonant modes through the phase matching condition (6). Figure 6(e,f) depict the radiation patterns of the cavity working in “On” and “Off” states, respectively. As expected, now our subwavelength cavity exhibit unidirectional radiations with narrowed half-power beam widths. The directivity is increased by more than 10.3 dB and cannot be inspected at other dips which do not correspond to highly-directive emissions.

## Discussion

In summary, we show that functionality-switching devices can be realized using carefully designed TMS with active PIN diodes incorporated. The underlying physics is that switching on/off the PIN diode can dynamically modify the reflection phases of the TMS by tuning the resonance frequency, and in turn, significantly changes those phase-related functionalities. Among many application possibilities, here we employed microwave experiments and FDTD simulations to demonstrate two examples: a helicity dynamical modulator for CP waves and a frequency-tunable subwavelength resonant cavity. Our studies setup a solid platform to design/realize phase-related functional devices in the microwave regime, such as the dynamically controlled anomalous reflectors and even animated holograms, and realizing these ideas would be interesting for future projects.

## Methods

### Experimental design, fabrication and measurements

The TMS is fabricated using the conventional print circuit board technique while the PIN diodes and inductors are attached to the top metallic microstructure using surface-mount technology with leads. The dielectric board is chosen as the F4B substrate with the permittivity of *ε*_*r*_ = 2.65, the thickness of *h* = 6 mm, and the loss tangent of 0.001. The metallic layers on both sides of the metasurface are copper with a thickness of 36 μm. All the SMT elements are checked by a multimeter of VICTOR VC9807A+ to guarantee the perfect electrical connection and the correct electrodes of the diodes. In the measurement setup, see Fig. S9 in the Supplementary Information, the GPD4303S from GWINSTEK is chosen as DC power and is connected to the two electrodes by the two thin wire. A pair of LP horn antennas (i.e., the source and receiver) are placed at the distance of 1.2 m from the sample to measure the reflection amplitude and phase. Their separation angle is set to be 10° to mimic the normal incidence case in our measurement. By simultaneously changing the orientations of the two horn antennas along x- or y- directions, four LP reflection coefficients are recorded through Agilent E8362C PNA vector network analyzer, which will be normalized against a reference signal reflected by the metallic plate with the same size of the TMS.

## Additional Information

**How to cite this article**: Xu, H.-X. *et al*. Dynamical control on helicity of electromagnetic waves by tunable metasurfaces. *Sci. Rep*. **6**, 27503; doi: 10.1038/srep27503 (2016).

## Supplementary Material

Supplementary Information
