# Supplementary material for: Dynamical control on helicity of electromagnetic waves by tunable metasurfaces
Source: Sci Rep. 2016 Jun 8;6:27503. doi: 10.1038/srep27503 (PMC4897613; doi:10.1038/srep27503)
Supplement: Supplementary Information [file srep27503-s1.pdf]

Supplementary Information

Dynamical control on helicity of electromagnetic waves by tunable metasurfaces

He-Xiu Xu[1,2]*, Shulin Sun[3], Shiwei Tang[1], Shaojie Ma[1], Qiong He[1,4], Guang-Ming Wang[2], Tong Cai[1,2], Hai-Peng Li[2] & Lei Zhou[1,4]*

[1]*State Key Laboratory of Surface Physics, Key Laboratory of Micro and Nano Photonic Structures (Ministry of Education) and Physics Department, Fudan University, Shanghai 200433, China*

[2]*Air and Missile Defense College, Air force Engineering University, Xi'an, 710051, China*

[3] *Shanghai Engineering Research Center of Ultra-Precision Optical Manufacturing, Green Photonics and Department of Optical Science and Engineering, Fudan University, Shanghai 200433, China*

[4]*Collaborative Innovation Center of Advanced Microstructures, Fudan University, Shanghai 200433, China*

[*]Corresponding authors: Lei Zhou, phzhou@fudan.edu.cn; He-Xiu Xu, hexiuxu@fudan.edu.cn.

1. Full control of the three resonances of TMS

To guide the designs of the TMS, we have performed a series of parametric analyses to achieve the full control of the three resonances (i.e., $f_1^{(y)}$, $f_2^{(y)}$ and $f_1^{(x)}$), as shown in Figs. S1-S5. Without loss of generality, the input EM wave is chosen as right-hand circular polarization (RCP).

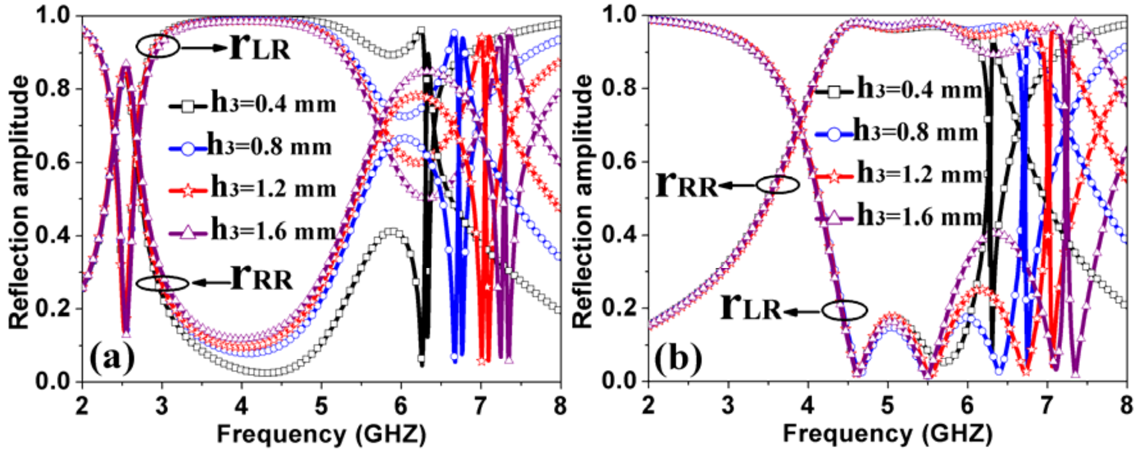

Figure S1. Effects of h_3 on the CP reflection coefficients of the TMS plate in the "On" (**a**) and "Off" (**b**) states.

Fig. S1 depicts the simulated circular polarization (CP) reflection coefficients $|r_{RR}|$ and $|r_{LR}|$ (co- and cross-polarization components) of the tunable metasurface (TMS) in the "On" and "Off" states as a function of split width h_3. In the "On" state (see Fig. S1 (a)), $|r_{RR}|$ increases significantly when h_3 increases from 0.4 mm to 1.6 mm in steps of 0.4mm. Therefore, the bandwidth for CP helicity conversion (i.e., Band I) shrinks and the in-band cross-polarization conversion efficiency also deteriorates. Moreover, we also notice that the performance of CP helicity hybridization in Band II around 6.3GHz has become worse as h_3 increased. However, in the "Off" state (see Fig. S1(b)), the resonance frequency $f_2^{(y)}$ shifts upwards significantly while the resonance frequencies $f_1^{(y)}$ and $f_1^{(x)}$ are almost fixed when h_3 increased, indicating an enlarged bandwidth of helicity conservation. Meanwhile, $|r_{RR}|$ fluctuates heavily, leading to the low CP purity (i.e. CP extinction ratio). Therefore, h_3 should

be cautiously designed to balance the performances of helicity conversion, hybridization, and conservation.

Fig. S2 illustrates the simulated CP reflection coefficients as a function of the gap length w_2. It is noted that, while w_2 increases from 3mm to 6mm, $|r_{RR}|$ and $|r_{LR}|$ almost are same in the "On" state. Meanwhile, the ripples of $|r_{RR}|$ and $|r_{LR}|$ become stronger in the "Off" state, implying the low CP extinction ratio. It is reasonable that $|r_{RR}|$ and $|r_{LR}|$ are almost immune to w_2 in the "On" state, because the capacity effect will break down after the gap is short-circuited by the PIN diode. In the "Off" state, although the gap capacitor will be enlarged with the increased w_2, it is still negligible to shift $f_1^{(y)}$ relative to the diode capacitance C_t and fringing capacitor C_f. Although $f_2^{(y)}$ and $f_1^{(x)}$ almost do not shift with the increase of w_2, their resonant intensity and in turn the reflection phase of the LP reflections are quite sensitive to w_2 , enhancing the fluctuation of reflection coefficients. As a consequence, w_2 should be as small as possible to gain an elegant performances of the TMS.

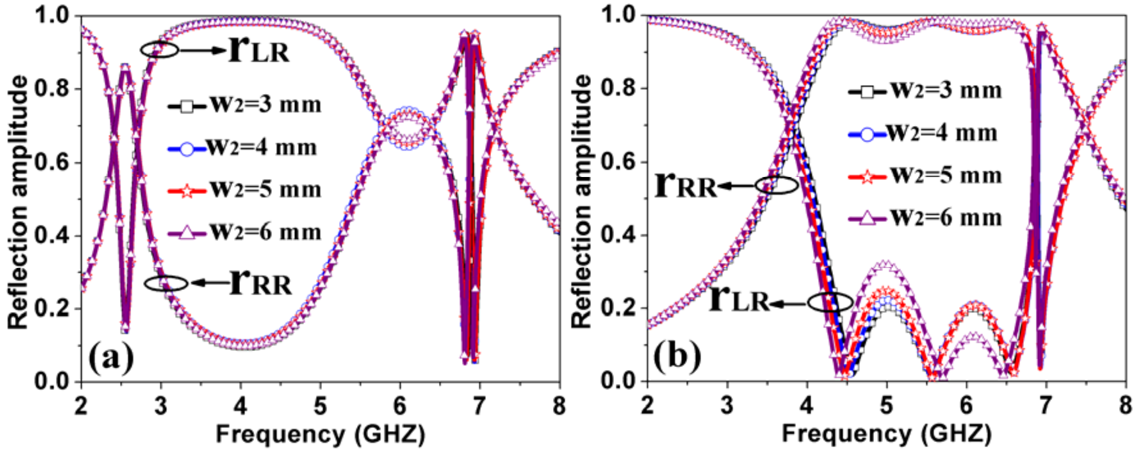

Figure S2. Effects of w_2 on the CP reflection coefficients of the TMS plate in the "On" (**a**) and "Off" (**b**) states.

Fig. S3 shows the simulated CP reflection spectra of the TMS with various radius R of circular ELC structure. It is predicable that $f_1^{(y)}$, $f_2^{(y)}$ and $f_1^{(x)}$ all move to lower frequencies as R increases in both "On" and "Off" state. However, the resonances frequencies $f_1^{(y)}$ and $f_1^{(x)}$

move toward each other while the resonance frequencies f_2 and f_3 move away from each other due to their distinct shift speeds. These issues will lead to the depressed performance of CP helicity conversion in the "On" state, while the varied in-band fluctuations of $|r_{LR}|$ and $|r_{RR}|$ in the "Off" state (i.e., smaller ripples between $f_1^{(y)}$ and $f_1^{(x)}$ while larger ripples between $f_2^{(y)}$ and $f_1^{(x)}$). Consequently, R should be elaborately designed by taking a trade off between the in-band performances in both states.

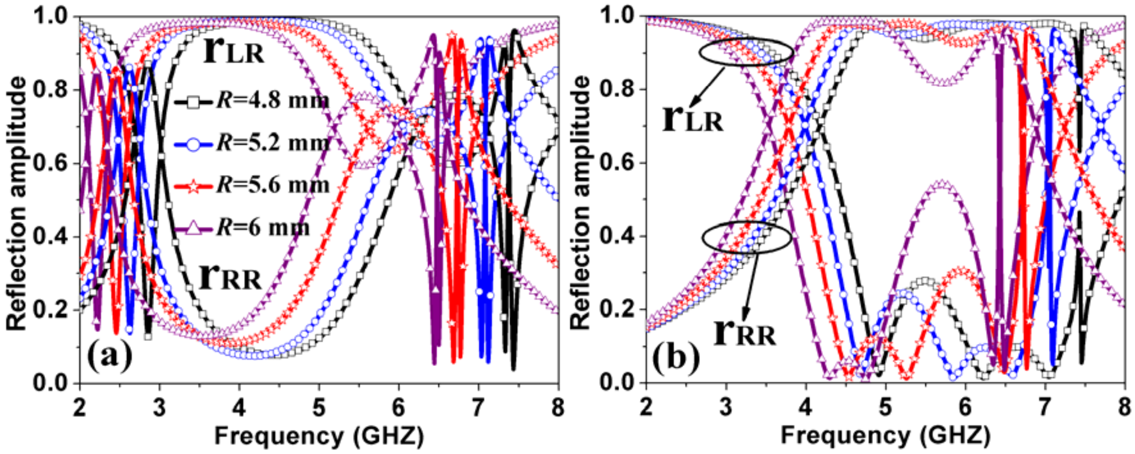

Figure S3. Effects of R on the CP reflection coefficients of the TMS plate in the "On" (**a**) and "Off" (**b**) states.

To provide an intuitive insight on the effect of the lumped inductor, we performed additional FDTD simulations by changing the inductance L_j of the SMT elements with the residual geometrical parameters kept as constant. As is illustrated in Fig. S4, the bandwidth of the CP helicity conversion and its extinction ratio in the "On" state deteriorates seriously as L_j increases from 5nH to 20nH in steps of 5nH. However, $|r_{RR}|$ around Band II increases significantly and finally approaches 100%, facilitating a fairly good CP helicity conservation performance while a worse CP helicity hybridization performance. In the "Off" state, the bandwidth of CP helicity conversion enlarges first and then shrinks as L_j increases. Therefore, a moderate L_j is chosen as about L_j=10nH in our practical sample, according to the available parameters of the SMT elements in the market.

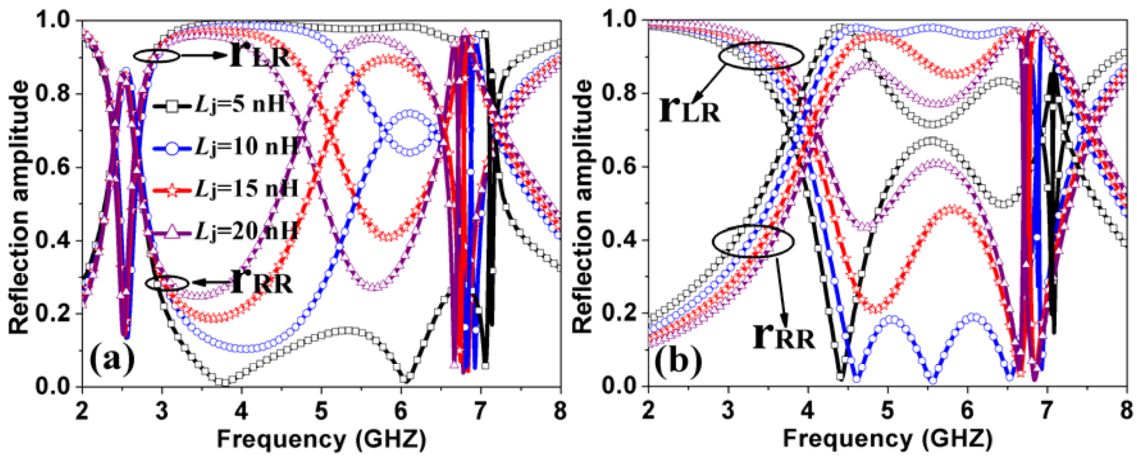

Figure S4. Effects of L_j on the CP reflection coefficients of the TMS plate in the "On" (**a**) and "Off" (**b**) states.

To clarify the tunable range of polarization modulation that the our TMS can achieve, the CP reflection coefficients $|r_{LR}|$ and $|r_{RR}|$ are calculated as a function of the junction capacitance C_j in the "Off" state (see Fig. S5). It is shown that $f_1^{(y)}$ shifts upwards first and then downwards while $f_2^{(y)}$ and $f_1^{(x)}$ are almost fixed when C_j increases from 0pF to 0.9pF. The red-shift of $f_1^{(y)}$ with reduced sensitivity seems to be saturated when C_j approaches 0.9pF. Moreover, the frequency band for CP helicity conservation are progressively divided into two independent ones with an interval band of CP helicity conversion. Therefore, C_j should be as small as possible to engineer a broad band for helicity conservation. Indeed, C_j can not be infinitely small and is restricted to values available in real manufactures. In the current design, we select C_j=0.15pF which guarantees a broadband CP helicity conservation and moderate frequency tuning range.

To be emphasized, since the resonance frequency $f_1^{(y)}$ of the TMS can only be tuned in quite a limited range (i.e., 3.15~3.9GHz) when C_j increases from 0.3pF to 0.9pF (the typical upper threshold of the varactor in the market), it is impossible to pursue a continuous modulation of EM wave polarization by gradually changing C_j. Fortunately, a resonance frequency switch between $f_1^{(y)}$=2.5GHz (while C_j=0pF) and $f_1^{(y)}$=4.6 GHz (while C_j=0.15pF) is large enough to design the dual-state TMS.

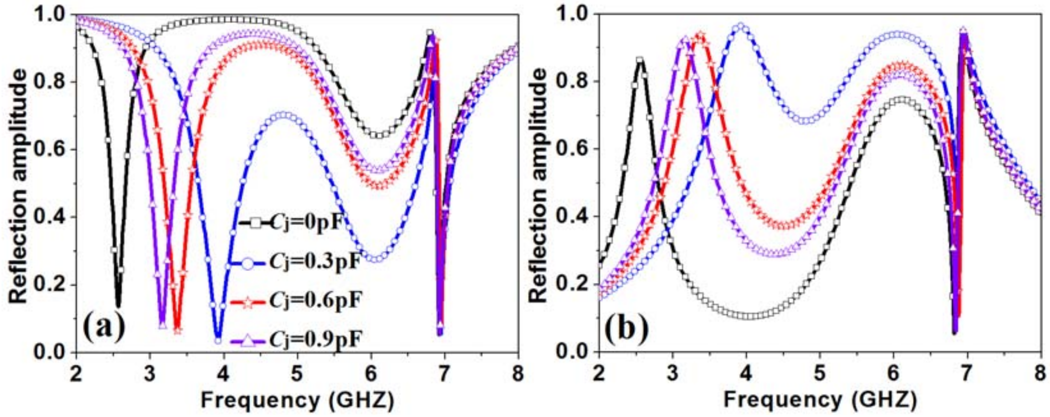

Figure S5. Effects of C_j on the CP reflection coefficients of the TMS plate. (**a**) $|r_{LR}|$ curve. (**b**) $|r_{RR}|$ curve.

2. Modeling of the TMS and diode

In Fig. S6, the PIN diodes are series connected lumped elements, which can be modeled as a series of L_s and R_s (L_s and C_j) in the "On" ("Off") state. Here, L_s represent the parasitic inductance, R_s is a total resistance composed of junction and ohmic resistance, and C_j is junction capacitance. In addition, C_s is the parasitic capacitance in package and can lead to a little larger C_t (total capacitance) than C_j in the "Off" state. In this particular design, L_s=0.7nH, C_j=0.15pF, C_t=0.18pF and R_s=2Ω. In the CMs of Fig. 2(b), the back metallic layer is represented by ground, whereas the localized magnetic response and the transmission effects through dielectric substrate (with impedance Zo and thickness h) were modeled by a transmission line (TL) with equivalent impedance Z_c and electrical length h_o for analysis convenience. According to TL theory, three resonances will occur with central frequencies determined by $f_1^{(y)} = 1/2\pi\sqrt{L_1C_1}$, $f_2^{(y)} = 1/2\pi\sqrt{L_2C_2}$ and $f_1^{(x)} = 1/2\pi\sqrt{L_3C_3}$, respectively. Supported by Fig. 4, i.e., $f_2^{(y)}$ originating from the resonance of two symmetric circular arcs and $f_1^{(y)}$ from that of the central wires ("Off" state) or central wire and arcs ("On" state), while $f_1^{(x)}$ from the thin bias line, now the contribution of the loop inductance and capacitance in CM are clear. They are $L_1 \approx L_w + L_s$ and $C_1 = 2C_p + C_f \approx C_f$ in "On" state, and are $L_1 \approx L_w$ and $C_1 \approx C_f * C_t/(C_f + C_t)$ in

“Off” state, respectively. Conversely, L_2≈L_a/2, C_2≈C_p and L_3≈L_j+L_{line}, C_3≈C_C in both states. Here, L_w and L_a are the inductors of the wire and circular arc, and C_p and C_f are the capacitors of the split and the parallel thin lines of two adjacent elements, and L_j and L_{line} are the inductors of SMT element and thin line, and C_C is the coupling capacitor of the splits between adjacent elements along x direction. The resonant losses of above three tanks are represented by R_1, R_2 and R_3, respectively.

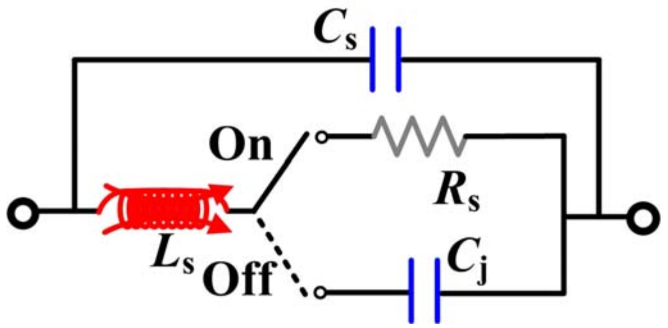

Figure S6. Equivalent circuit models of the PIN diode.

3. Polarization extinction ratio of the TMS

The polarization extinction ratio σ is parameter to evaluate the CP purity of reflected beam which can be defined as $\sigma = 20\log_{10}(|r_{LR}|/|r_{RR}|)$. Fig. S7 shows the simulated and measured σ to visualize the CP helicity modulations at different frequencies. In the On” state, the device “functions as a CP helicity convertor with simulated (measured) $|r_{RR}|$<0.31 ($|r_{RR}|$<0.29), $|r_{LR}|$>0.92 ($|r_{LR}|$>0.9) and σ>10.2dB within 2.9~5.13GHz (3.11~5.01 GHz), and a CP helicity hybridizer with σ<3dB within 5.58~6.69 GHz (5.51~6.94 GHz). However, the device in “Off” state behaves as a CP helicity retainer with $|r_{LR}|$<0.3 ($|r_{LR}|$<0.32), $|r_{RR}|$>0.94 ($|r_{RR}|$>0.87) and σ<-10 dB within 4.33~6.63GHz (4.21~7.01 GHz).

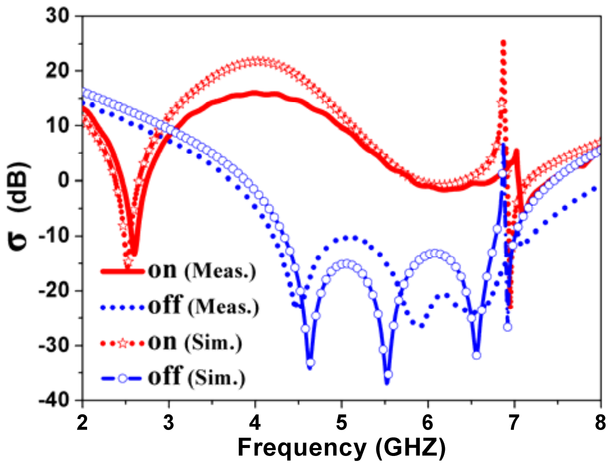

Figure S7. Simulated and measured polarization extinct ratio of the TMS plate in the "On" and "Off" state.

4. Additional results for Figure 5

To understand the deviations between the simulated and measured reflection phase around $f_2^{(y)}$ (see Figs. 3 and 5 in the main text), we have performed more FDTD simulations to investigate the influence of the resistance R_j of the SMT elements on the reflection phase. Such effect can be well explained by the couple mode theory (CMT)[1-3], based on which the complex reflection coefficient r of the metasurface can be derived as:

$$r = -1 + \frac{2/\tau_r}{-i(\omega - \omega_0) + 1/\tau_a + 1/\tau_r}. \qquad \text{(S1)}$$

Here, τ_a and τ_r are the lift times of the resonance due to absorption inside the structure and radiation to the far field, respectively. According to CMT, the absorptive quality factor $Q_a = \omega_0 \tau_a / 2$ and the radiation quality factor $Q_r = \omega_0 \tau_r / 2$, retrievable from the simulated or measured reflection spectra, will collectively determine the metasurface to be either a EM absorber or a EM phase modulator.

Here, we study the influence of R_j on the reflection phase of TMS based on FDTD simulations. As shown in Fig. S8(a), the reflection phase undergo a continuous variation from -180° to 180° while R_j increases from 0 Ω to 40 Ω in the "On" state. However, if R_j increases

to 60 Ω or 80 Ω, the reflection phase can only cover a range of less than 90°. Such behaviors are consistent with the Q diagrams (Fig. S8(c)), showing that Q_r and Q_a are located in the under-damped region (above red dashed curve) when R_j=0, 20 and 40Ω, while in the over-damped region (under red dashed curve) when R_j=60 and 80 Ω. Similar arguments are also valid in the "Off" state. As shown in Figs. S8(b) and S8(d), the TMS is located in the under-damped region showing a full range phase (i.e., 360°) modulation as R_j=0 Ω, while in the over-damped region showing a limited phase modulation as R_j=20, 40, 60 and 80 Ω. Therefore, the inconsistency between the simulated and measured reflection phase around f_2 may attribute to the increased R_j in real fabricated sample. To demonstrate this argument, we have retrieved the absorptive quality factor ($Q_a = 31.9\Omega$) and the radiation quality factor ($Q_r = 339.2\Omega$) according to the measured reflection spectra, indicating that the TMS is located in the over-damped region (i.e., $Q_a < Q_r$) and thus supplies a limited reflection phase region.

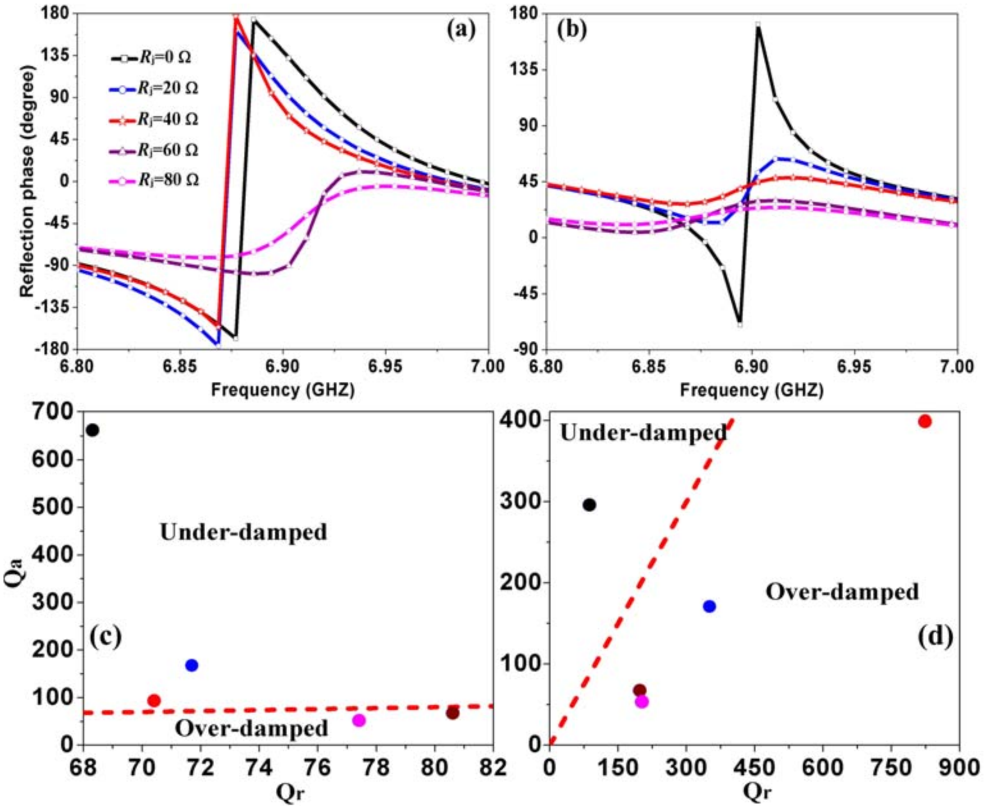

Figure S8. The reflection phases (**a, b**) and the calculated $Q_a \sim Q_r$ phase diagrams (**c, d**) of the TMS plate in the "On" (**a, c**) and "Off" (**b, d**) states. The dash line in (**c**) and (**d**) correspond to that of $Q_a = Q_r$.

5. Experimental setup for reflection measurements

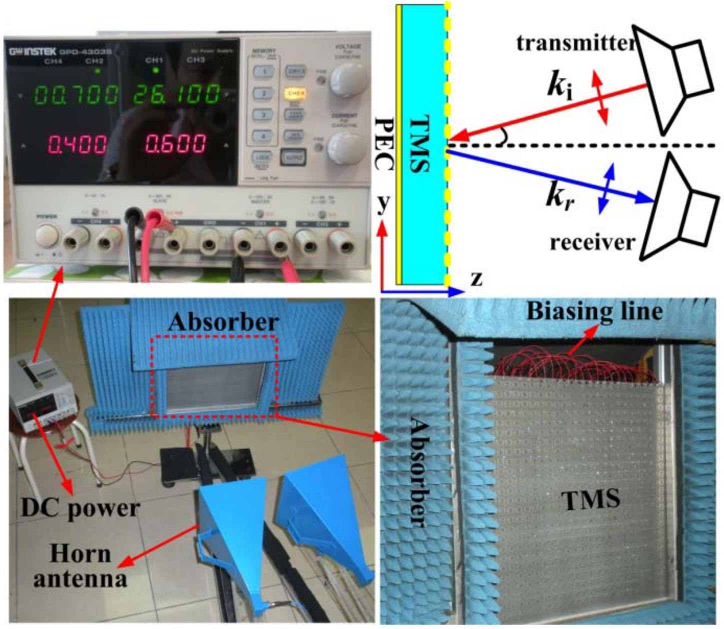

Figure S9. Illustration of the reflection measurement setup. All cathode and anode conducting wires from the TMS plate eventually pooled in the two electrodes of the DC power. A mass of absorbing materials are utilized aside the TMS sample to fully eliminate the reflections from surrounding objects.

References

1. Haus, H. A. *Waves and Fields in Optoelectronics*, Englewood Cliffs. NJ: Prentice-Hall, **1984**.
2. Fan, S., Suh, W. & Joannopoulos, J. D. Temporal coupled-mode theory for the Fano resonance in optical resonators. J. Opt. Soc. Am. A **20**, 569 (2003).
3. Suh, W., Wang, Z. & Fan, S. Temporal coupled-mode theory and the presence of non-orthogonal modes in lossless multimode cavities. IEEE J. Quantum Electron. **40**, 1511 (2004).
